# Supplementary material for: Patterns of Intron Gain and Loss in Fungi
Source: PLoS Biol. 2004 Nov 30;2(12):e422. doi: 10.1371/journal.pbio.0020422 (PMC532390; doi:10.1371/journal.pbio.0020422)
Supplement: Table S1 — Also available at http://genes.mit.edu/NielsenEtAl/. (4.3 MB ZIP). [file pbio.0020422.st001.zip › NielsenEtAl/html/547.html]

AN3070.1.NCU09709.1.MG03857.1.FG06313.1


```
 CLUSTAL W (1.82) Multiple Sequence Alignments - Introns Inserted


Sequence 1: NCU09709.1	544 aa
Sequence 2: FG06313.1	546 aa
Sequence 3: MG03857.1	544 aa
Sequence 4: AN3070.1	539 aa
Alignment Length: 546 aa
Number Identitical Residues: 410 aa
Alignment Score (without introns) 16975


MG03857.1 	MSAAQLLNPKAESR0RRGEALRVNISAGEGLQDVLKSNLGPLGTIKM2LVDGSGQ0IKLT
NCU09709.1	MSAAQLLNPKAESR0RRGEALKVNITAGEGLQDVLKSNLGPSGTIKM2LVDGAGQ0IKLT
FG06313.1 	MSAAQLLNPKAESR0RRGEALKVNISAGEGLQDVLKSNLGPRGTIKM2LVDGAGQ0IKLT
AN3070.1  	MSATQLLNPKAESR0RRAEALKVNINAGEGLQDVLKSNLGPSGTLKM2LVDGAGG0IKLT
          	***:********** **.***:***.*************** **:** ****:*  ****

MG03857.1 	KDGNVLLREM~QIQNPTA~VMIARAATAQDDICGDGTTSVVLLVGELLKQAERHIQEGLH
NCU09709.1	KDGNVLLREM~QIQNPTA~VMIARAATAQDDICGDGTTSVVLLVGELLKQADRYISEGLH
FG06313.1 	KDGNVLLREM~QIQNPTA~VMIARAATAQDDICGDGTTSVVMLVGELLKQADRYISEGLH
AN3070.1  	KDGNVLLREM0QIQNPTA0VMIARAATAQDDITGDGTTSVVLLVGELLRQADRHISEGLH
          	********** ******* ************* ********:******:**:*:*.****

MG03857.1 	PRIIADGFEIAKNEALK0FLDDFRIPRDIDRELLISVARTSLATKLNSTLANKLTPDIVD
NCU09709.1	PRIITDGFEIAKTEALK0FLDQFKLPREVDRELLLSVARTSLATKLSASLAQSLTADIVD
FG06313.1 	PRIITDGFEVAKIEALK0FLDSFKLAKEVDRELLLSVARTSLTTKLNATLAQKLTPDIVD
AN3070.1  	PRVITDGYEIAKNEALK0FLDKFKIERAIDRELLLSVARTSLSTKLNSALAEKLTPDIVD
          	**:*:**:*:** **** ***.*:: : :*****:*******:***.::**:.**.****

MG03857.1 	AVLAIYKAPAKPDLHMVEIMKMQHRTASDTQLIRGLVLDHGARHPDMPKRLENAYILTLN
NCU09709.1	AVLAIYQPPAKPDLHMIEIMTMQHRTASDTQLIRGLALDHGARHPDMPKRVENAYILTLN
FG06313.1 	AVLAIYQEGAKPDLHMVEIMKMQHRTAADTRLIRGLALDHGARHPDMPKRLENCYILTMN
AN3070.1  	AVLAIHRAPEKPDLHMVEIMTMQHRTSSDTQLIRGLALDHGARHPDMPKRVENAFILTLN
          	*****::   ******:***.*****::**:*****.*************:**.:***:*

MG03857.1 	VGLEYEKSEINSGFFYNSAEQRDKLVESERRHVDAKLKKIVELKKEVCGNDPKKSFVIVN
NCU09709.1	VSLEYEKSEINSGFFYSSAEQRDKLVESERRFVDAKLKKIVELKKEVCGNDPTKNFVIIN
FG06313.1 	VSLEYEKTEINSSFFYSSAEQRDKLVESERRFVDAKLKKIVDLKKELCGNDGTKNFVVIN
AN3070.1  	VSLEYEKSEINSGFYYSSAEQRDKLVESERKFVDAKLQKIVELKKQVCGDDPKKGFVVIN
          	*.*****:****.*:*.*************:.*****:***:***::**:* .*.**::*

MG03857.1 	QKGIDPLSLDVLAKNGIFALRRAKRRNMERLQLIAGGVAQNSVEDLSPEVLGWAGLVYEQ
NCU09709.1	QKGIDPLSLDVLAKNGILALRRAKRRNMERLQLVCGGVAQNSVDDLSPEVLGWAGLVYEQ
FG06313.1 	QKGIDPLSLDVLAKNNILALRRAKRRNMERLQLVCGGVAQNSVDDLSEEVLGYAGLVYEQ
AN3070.1  	QKGIDPLSLDVLVKNGIMALRRAKRRNMERLQLICGGTAQNSVEDLSPEVLGWAGLVYEH
          	************.**.*:***************:.**.*****:*** ****:******:

MG03857.1 	QLGEEKYTFIEEVKDPKSVTLLIKGPNAHTITQITDAVRDGLRSVYNTIVDKSVVPGGGA
NCU09709.1	QLGEEKYTFIEEVKDPKSVTLLIKGPNAHTITQLKDAVRDGLRSVYNMIVDKSVVPGGGA
FG06313.1 	TLGEEKYTFVEEVKDPKSVTLMIKGPNAHTIAQVTDAVRDGLRSVYNMIVDKSVVPGAGA
AN3070.1  	QLGEEKYTFVEEVKDPKSVTILIKGPNGHTIAQVKDAVRDGLRSVYNTIVDGCVIPGAGA
          	 ********:**********::*****.***:*:.************ *** .*:**.**

MG03857.1 	FQVACAAHLRSDAFSKTVKGKGKWGVQAFADALLIIPKTLAANAGHDIQESL1ANLQDDL
NCU09709.1	FQVACAAHLKSDAFTKTVKGKAKWGVEAFADALLVIPKTLAANAGLDVQDSL1AALQDEQ
FG06313.1 	FQVACASHLKSDAFGKTVKGKAKWGVEAFADALLIIPKTLAANAGLDIQDAL1ADLQDEY
AN3070.1  	FQVACHAHLTSDSFRKSVKGKARWGVEAFADALLVIPKTLADNSGHDIQDSL~ALLQEEQ
          	***** :** **:* *:****.:***:*******:****** *:* *:*::* * **:: 

MG03857.1 	ADGNAFGLDLQTGGTMDPTLEGVYDSFRVLRNSVASSASIASNLLLCDEMLKARQMGRQG
NCU09709.1	AEGNVVGLDLATGEPMDPTLEGVYDSFRVLRNCVASSAGIASNLLLCDELLKARQMGRAG
FG06313.1 	ADGNVVGLNLETGEPMDPELEGIYDSYRVLRNCIASSSSIASNLLLCDELLKARQMGRAG
AN3070.1  	ADGNVVGLDLTTGEPMDPVQEGVFDSFRVLRNCIASSTGIASNLLLCDELLKARQMGKQG
          	*:**..**:* ** .***  **::**:*****.:***:.**********:*******: *

MG03857.1 	GPGPGMDGPEE--
NCU09709.1	GPGPGMDGPDS--
FG06313.1 	GPGPGMDGPNDHM
AN3070.1  	GPG-GME------
          	*** **:
```
